# A Rho GDP Dissociation Inhibitor Produced by Apoptotic T-Cells Inhibits Growth of *Mycobacterium tuberculosis*

**DOI:** 10.1371/journal.ppat.1004617

**Published:** 2015-02-06

**Authors:** Sambasivan Venkatasubramanian, Rohan Dhiman, Padmaja Paidipally, Satyanarayana S. Cheekatla, Deepak Tripathi, Elwyn Welch, Amy R. Tvinnereim, Brenda Jones, Dan Theodorescu, Peter F. Barnes, Ramakrishna Vankayalapati

**Affiliations:** 1 Department of Pulmonary Immunology, Center for Biomedical Research, University of Texas Health Center, Tyler, Texas, United States of America; 2 Keck School of Medicine, University of Southern California, Los Angeles, California, United States of America; 3 University of Colorado Comprehensive Cancer Center, Aurora, Colorado, United States of America; Portland VA Medical Center, Oregon Health and Science University, UNITED STATES

## Abstract

In this study, we found that a subpopulation of CD4^+^CD25^+^ (85% Foxp3^+^) cells from persons with latent tuberculosis infection (LTBI) inhibits growth of *M. tuberculosis* (*M. tb*) in human monocyte-derived macrophages (MDMs). A soluble factor, Rho GDP dissociation inhibitor (D4GDI), produced by apoptotic CD4^+^CD25^+^ (85% Foxp3^+^) cells is responsible for this inhibition of *M. tb* growth in human macrophages and in mice. *M. tb*-expanded CD4^+^CD25^+^Foxp3^+^D4GDI^+^ cells do not produce IL-10, TGF-β and IFN-γ. D4GDI inhibited growth of *M. tb* in MDMs by enhancing production of IL-1β, TNF-α and ROS, and by increasing apoptosis of *M. tb*-infected MDMs. D4GDI was concentrated at the site of disease in tuberculosis patients, with higher levels detected in pleural fluid than in serum. However, in response to *M. tb*, PBMC from tuberculosis patients produced less D4GDI than PBMC from persons with LTBI. *M. tb*-expanded CD4^+^CD25^+^ (85% Foxp3^+^) cells and D4GDI induced intracellular *M. tb* to express the dormancy survival regulator DosR and DosR-dependent genes, suggesting that D4GDI induces a non-replicating state in the pathogen. Our study provides the first evidence that a subpopulation of CD4^+^CD25^+^ (85% Foxp3^+^) cells enhances immunity to *M. tb*, and that production of D4GDI by this subpopulation inhibits *M. tb* growth.

## Author Summary

Most people who are infected with *Mycobacterium tuberculosis* (*M. tb*) have latent tuberculosis infection (LTBI) with protective immunity. Patients with active tuberculosis have severe disease and ineffective immunity. Understanding how LTBI individuals control infection without developing disease provides important insight into the mechanisms of protective immunity against tuberculosis, and this information is essential for development of an effective vaccine. It is known that a lymphocyte population called T-cells contributes significantly to protective immunity against tuberculosis infection. In the current study, using human and murine models of *M. tb* infection, we found that a soluble factor, Rho GDP dissociation inhibitor (D4GDI), produced by a subpopulation of T-cells (CD4^+^CD25^+^Foxp3^+^) inhibits *M. tb* growth. We also found that D4GDI induces *M. tb* genes that are expressed during the non-replicative state. Our results suggest that D4GDI has a previously undescribed positive effect on immunity by enhancing host antimicrobial activity. These findings also may aid in understanding the factors that induce LTBI. Further, this information will facilitate development of improved vaccines and immunotherapeutic strategies to prevent and treat tuberculosis, respectively.

## Introduction

Tuberculosis (TB) causes an estimated 1.7 million deaths world-wide annually. Reducing morbidity and mortality from TB hinges on developing an improved vaccine, which in turn depends on understanding the immune response. T cells play a crucial role in protective immunity against *Mycobacterium tuberculosis (M. tb)* and other intracellular pathogens [1] in part through production of IFN-γ, which is required for resistance to infection [2]. However, uncontrolled T-cell responses can cause tissue damage, which can be reduced by regulatory CD4^+^ T-cells (Tregs) that express CD25 and Foxp3 [3].

It is generally believed that CD4^+^CD25^+^Foxp3^+^ T-cells inhibit effective immunity to microbial pathogens. CD4^+^Foxp3^+^ T-cells accumulate at sites of infection [4] and prevent efficient clearance of infection in mice infected with *M. tb* [5]. Recently we found that Tregs expand in response to *M. tb* in healthy tuberculin reactors and that *M. tb* mannose-capped lipoarabinomannan converts some CD4^+^CD25^-^Foxp3^-^ cells to CD4^+^CD25^+^Foxp3^+^ cells [6]. We also found that the programmed death-1 receptor (PD-1) and cytokine inducible SH2-containing protein (CISH) control expansion of *M. tb*-induced Tregs [7]. Furthermore, human CD4^+^CD25^+^Foxp3^+^ cells produce TGF-β and IL-10, and inhibit IFN-γ production by CD4^+^ and CD8^+^ cells [6], suggesting that they may limit tissue inflammation and destruction. Other studies have found increased number of CD4^+^Foxp3^+^ T-cells in TB patients, which inhibit immune responses [6], [8], [9].

However, in humans, some activated T-cells express Foxp3 transiently and these cells lack classical regulatory function [10]–[12]. In the current report, we found that *M. tb*-activated CD4^+^CD25^+^ (85% Foxp3^+^) T-cells from individuals with latent tuberculosis infection (LTBI) can inhibit growth of *M. tb* in human monocyte-derived macrophages (MDMs) through production of Rho GDP dissociation inhibitor (D4GDI), a small GTP-binding protein. In addition, D4GDI reduced the bacillary burden in mice infected with *M. tb.* These findings demonstrate that human CD4^+^CD25^+^ (85% Foxp3^+^) T-cells can contribute to immune defenses by enhancing antimicrobial activity.

## Results

### CD4^+^CD25^+^ (85% Foxp3^+^) cells inhibit growth of *M. tb* in MDMs

**Figure 1:**
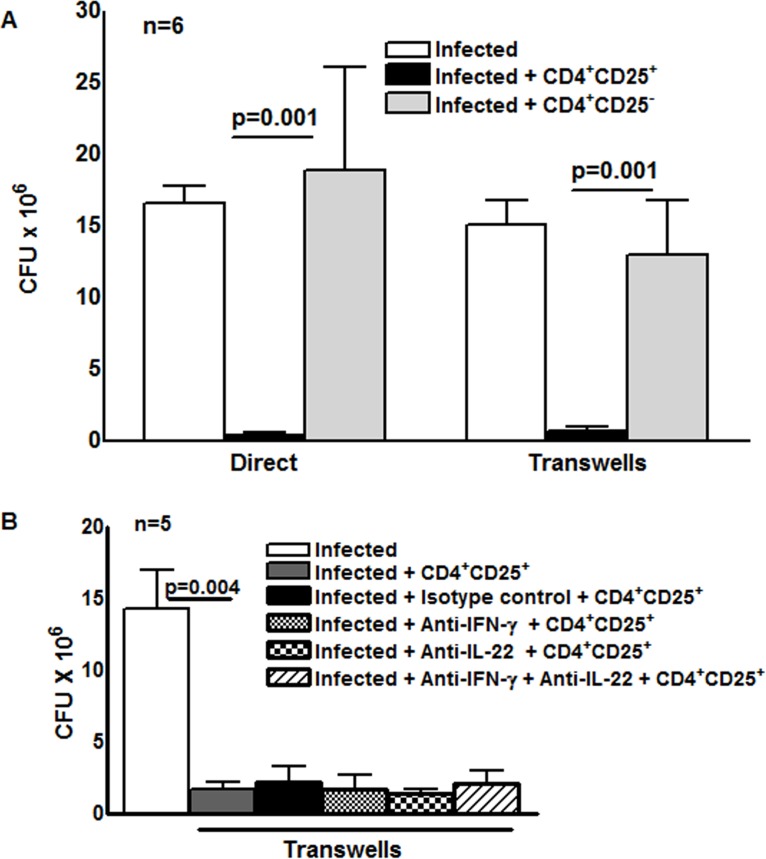
Effect of CD4^+^CD25^+^Foxp3^+^cells on *M. tb* growth. (A) Freshly isolated CD4^+^ cells and CD14^+^ monocytes from 6 individuals with LTBI were cultured with γ-irradiated *M. tb* H37Rv. After 4 days, CD4^+^CD25^+^ (85–90% Foxp3^+^) and CD4^+^CD25^-^ (<5% Foxp3^+^) cells were isolated, as outlined in the methods. MDMs from the same donors were infected with H37Rv at a MOI of 1:2.5 (2.5 *M. tb* to one MDM). After 2 hr MDMs were washed to remove extracellular bacteria and *M. tb*-expanded CD4^+^CD25^+^ (85–90% Foxp3^+^) or CD4^+^CD25^-^ (<5% Foxp3^+^) cells were cultured in Transwells, or directly in the same wells, at a T-cell:macrophage ratio of 1:9. After 2 hr and 7 days, CFU were measured and mean values, p values and SEs are shown. In 6 individuals with LTBI the number of CFU after 2 hr was 1.2 ± 0.3 × 10^6^. (B) H37Rv infected MDMs and *M. tb*-expanded CD4^+^CD25^+^ (85–90% Foxp3^+^) cells in Transwells were cultured as in panel A. To some wells, anti-IFN-γ (10 μg/ml), anti-IL-22 (10 μg/ml), alone or together (5 + 5 μg/ml), or isotype control antibodies (10 μg/ml) were added on days 0 and 3. After 7 days, CFU were measured and data for 5 individual LTBI+ donors was shown. Mean values, p values and SEs are shown.

**Figure 2:**
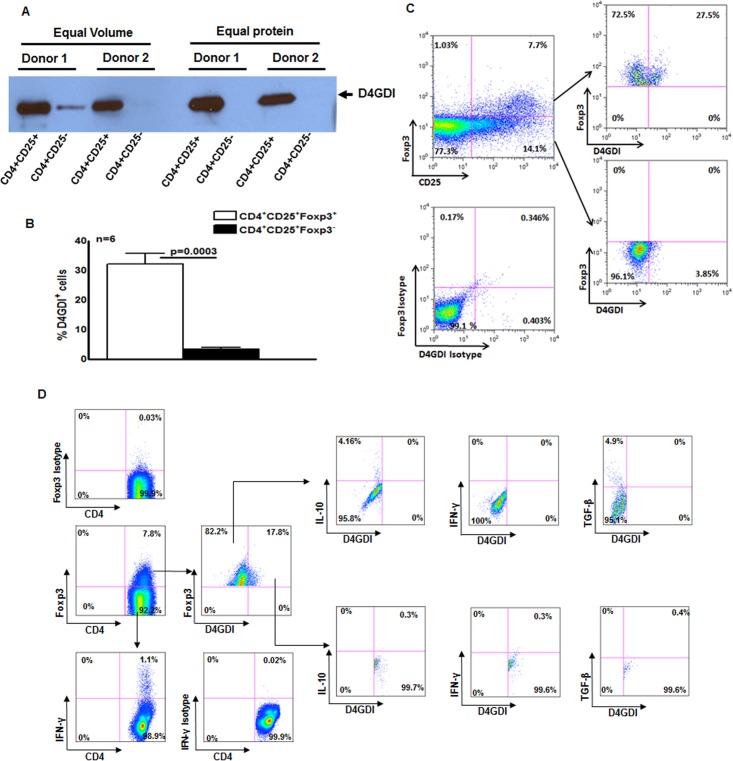
A subset of Foxp3^+^ cells express D4GDI. (A) CD4^+^ cells from PBMC of 6 donors with LTBI were cultured with autologous monocytes and γ-irradiated *M. tb* (10 μg/ml). After 4 days, CD4^+^CD25^+^ (85–90% Foxp3^+^) and CD4^+^CD25^‑^ (<5% Foxp3^+^) cells were isolated, and cultured overnight in serum-free medium. Culture supernatants were subjected to Western blotting with anti-D4GDI. Data from 2 individuals with LTBI are shown. (B) CD4^+^ cells and autologous monocytes from 6 donors were cultured as in panel A. After 4 days, the percentages of D4GDI^+^ cells in CD4^+^Foxp3^+^ and CD4^+^Foxp3^‑^ cells were determined by flow cytometry. Mean values, p values and SEs are shown. (C) A representative flow cytometry plot is shown. (D) PBMC from 5 individuals with LTBI were cultured with γ-irradiated *M. tb* H37Rv. After 4 days, the percentages ofCD4+Foxp3+D4GDI+IFN-γ+, CD4+Foxp3+D4GDI+IL-10+ and CD4+ Foxp3+D4GDI+TGF-β+ cells were determined by flow cytometry. A representative flow cytometry plot is shown.

To determine the effect of *M. tb*-activated CD4^+^CD25^+^Foxp3^+^ cells on intracellular mycobacterial growth, freshly isolated CD4^+^ cells and CD14^+^ monocytes from 6 LTBI were cultured with γ-irradiated *M. tb* H37Rv. After 4 days, CD4^+^CD25^+^ (85–90% Foxp3^+^) and CD4^+^CD25^-^ (<5% Foxp3^+^) cells were isolated, as outlined in the methods. MDMs from the same donors were infected with *M. tb* H37Rv at a MOI of 1:2.5, as detailed in the methods, and *M. tb*-expanded CD4^+^CD25^+^ (85% Foxp3^+^) or CD4^+^CD25^-^Foxp3^-^ cells were cultured in Transwells, or directly in the same wells containing infected macrophages. On day “0” (2 hr after infection) the number of CFU per well were 1.2 ± 0.38× 10^6^. After 7 days, 16.5 ± 1.2 × 10^6^ CFU per well were present in MDMs cultured without T-cells. Addition of CD4^+^CD25^+^ (85% Foxp3^+^) cells to the same well or in Transwells reduced CFU to 0.4 ± 0.2 × 10^6^ (>95% inhibition, p = 0.001 for both comparisons, Fig. 1A). Addition of CD4^+^CD25^-^Foxp3^-^ cells had no effect (Fig. 1A). This surprising result indicates that soluble factors produced by CD4^+^CD25^+^ (85% Foxp3^+^) cells strongly inhibit *M. tb* H37Rv growth in macrophages. This finding was unlikely to be due to contamination of CD4^+^CD25^+^ cells with activated CD4^+^ cells because very few cells expressed IFN-γ (Fig. 2D). Furthermore, we added a very low ratio of T-cells to macrophages (1:9), whereas published data indicate that much higher ratios of activated CD4^+^ cells are needed to reduce *M. tb* growth by 50–80% [13], [14]. Even though we used low numbers of CD4^+^CD25^-^Foxp3^+^cells for the above study, it is possible that the effects seen in the above experiment may be due to small numbers of contaminating IFN-γ- or IL-22-producing activated T-cells or NK cells. However, in 5 LTBI+ individuals, when CD4^+^CD25^+^Foxp3^+^ cells were cultured in Transwells with *M. tb*-infected MDMs, addition of anti-IFN-γ or anti-IL-22, alone or together, did not reduce inhibition of *M. tb* growth in MDMs by CD4^+^CD25^+^ (85% Foxp3^+^) cells (Fig. 1B).

### *M. tb*-expanded CD4^+^CD25^+^ (85%Foxp3^+^) cells produce D4GDI

To identify the soluble factor/s produced by *M. tb*-expanded CD4^+^CD25^+^Foxp3^+^ cells that inhibit *M. tb* H37Rv growth in macrophages, CD4^+^ cells and autologous monocytes from 6 persons with LTBI were cultured with γ-irradiated *M. tb* H37Rv. After 4 days, CD4^+^CD25^+^Foxp3^+^ and CD4^+^CD25^-^Foxp3^-^ cells were isolated by immunomagnetic sorting, and cultured overnight in serum-free medium. The supernatants were concentrated and proteins in the supernatants were resolved by 2D gel electrophoresis. Only supernatants from CD4^+^CD25^+^Foxp3^+^ cells showed strong expression of a protein that was identified as D4GDI (100% match by LC MS/MS analysis, S1 Fig.).

We next found that D4GD1 was abundant in supernatants from *M. tb*-expanded CD4^+^CD25^+^ (85% Foxp3^+^) cells but not those from CD4^+^CD25^-^Foxp3^-^ cells, using Western blotting (Fig. 2A). As additional confirmation, we isolated CD4^+^ cells from 6 individuals with LTBI, and cultured them with autologous monocytes and γ-irradiated *M. tb* for 4 days. We found that 33 ± 4% of CD4^+^Foxp3^+^ cells were D4GDI^+^, compared to only 3.2 ± 0.7% of CD4^+^CD25^+^Foxp3^-^ cells (p = 0.0003, Figs. 2B and C). This suggests that a subpopulation of CD4^+^Foxp3^+^ cells produce D4GDI. In 5 individuals with LTBI, we measured IL-10, TGF-β and IFN-γ-positive CD4^+^CD25^+^Foxp3^+^D4GDI^+^ cells in γ-irradiated *M. tb*-stimulated PBMC. As shown in Fig. 2D, CD4^+^CD25^+^Foxp3^+^D4GDI^-^ but not CD4^+^CD25^+^Foxp3^+^D4GDI^+^ cells are the source for IL-10 (5 ± 1.2% vs. 0.7 ± 0.3%, p = 0.02) and TGF-β (3.6 ± 2.1% vs. 0.5 ± 0.5%, p = 0.01). CD4^+^CD25^+^Foxp3^-^ cells but not CD4^+^CD25^+^Foxp3^+^D4GDI^-^ cells or CD4^+^CD25^+^ Foxp3^+^D4GDI^+^ cells are the major source or IFN-γ (1.2 ± 0.2% vs. 0.4 ± 0.2%, and 0.64 ± 0.1%, p = 0.06).

### D4GDI inhibits growth of *M. tb* in human macrophages

**Figure 3:**
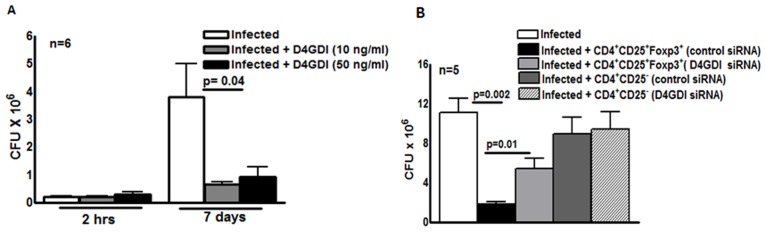
D4GDI inhibits growth of *M. tb* in macrophages. (A) Recombinant D4GDI inhibits growth of *M. tb*. MDMs from 6 LTBI+ individuals were infected with H37Rv at a MOI of 1:2.5 (2.5 *M. tb* to one MDM), with or without recombinant D4GDI. After 7 days, CFU were measured. Mean values, p values and SEs are shown for CFU per well. (B) D4GDI siRNA reverses Treg-dependent *M. tb* growth inhibition in macrophages. CD4^+^ cells from PBMC of 5 donors with LTBI+ were transfected with siRNA for D4GDI or scrambled siRNA, then cultured with autologous monocytes and γ-irradiated *M. tb* (10 μg/ml). After 4 days, CD4^+^CD25^+^ (85–90% Foxp3^+^) and CD4^+^CD25^-^ (<5% Foxp3^+^) cells were isolated, and cultured in Transwells with MDMs infected with H37Rv at a MOI of 1:2.5. After 7 days, CFU were measured. Mean values, p values and SEs are shown.

We next wished to determine if D4GDI inhibits *M. tb* H37Rv growth in human MDMs. Recombinant D4GDI had no effect on uninfected MDM viability (S2 Fig.). Recombinant D4GDI reduced CFU in MDMs by 75–84% (p = 0.04, Fig. 3A). Next, we treated *M. tb*-expanded CD4^+^CD25^+^ (85% Foxp3^+^) cells with D4GDI siRNA, which inhibited D4GDI mRNA expression by 70% to 80%, as quantified by real-time PCR. In contrast, D4GDI siRNA did not affect Foxp3 expression. In 5 donors with LTBI, CD4^+^CD25^+^ (85% Foxp3^+^) cells treated with scrambled siRNA reduced *M. tb* growth in MDMs by 84% (p = 0.002, Fig. 3B). In contrast, CFU were 3-fold higher in MDMs exposed to D4GDI siRNA-transfected CD4^+^CD25^+^Foxp3^+^ cells (p = 0.01, Fig. 3B). CD4^+^CD25^-^ cells treated with D4GDI or scrambled siRNA did not reduce CFU in MDMs. These findings indicate that D4GDI produced by *M. tb*-expanded CD4^+^CD25^+^ (85% Foxp3^+^) T-cells inhibits intracellular growth of *M. tb*.

### D4GDI inhibits growth of *M. tb* in mice

**Figure 4:**
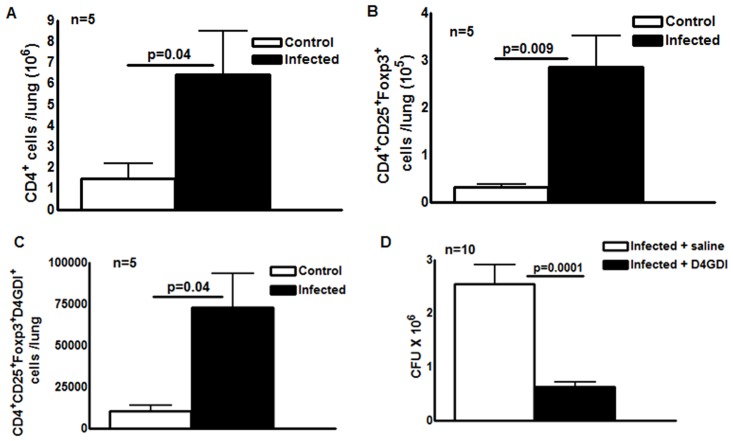
D4GDI inhibits growth of *M. tb* in mice. Ten wild type C57BL/6 mice were infected with 50–100 CFU of H37Rv by aerosol. Five mice were sacrificed after 24 hr and the bacterial burden in lungs was confirmed to be 50–100 CFU. The remaining five mice were sacrificed after 30 days. Total number of (A) CD4^+^ cells (B) CD4^+^CD25^+^Foxp3^+^ cells (C) CD4^+^CD25^+^Foxp3^+^D4GDI^+^ cells in the lungs of uninfected and *M. tb*-infected mice were measured by flow cytometry. (D) Twenty C57BL/6 wild type mice were infected with 50–100 CFU of *M. tb* H37Rv by aerosol. Ten infected mice were given recombinant D4GDI (10ng/ml) through tail vein injection on day 0, 4, 8, 12, 16, 20, 24 and 28 after infection. Thirty days later, bacterial burden in the lungs was measured by plating lung homogenates and counting CFU. Mean values, p values and SEs are shown.

Our data in humans (Fig. 3) suggest that CD4^+^CD25^+^ (85% Foxp3^+^) cells inhibit growth of *M. tb* through production of D4GDI. To determine if these cells express D4GDI during *M. tb* infection in vivo, we infected C57BL/6 mice with *M. tb* H37Rv. After 30 days, we quantified CD4^+^CD25^+^Foxp3^+^D4GDI^+^ cells in the lungs of uninfected and *M*. *tb* H37Rv-infected mice. Immunolabeling and flow cytometry showed that the total number of CD4^+^ cells increased 4.5 fold (p = 0.04, Fig. 4A), CD4^+^CD25^+^Foxp3^+^ cells increased around 9 fold (p = 0.009, Fig. 4B) and CD4^+^CD25^+^Foxp3^+^D4GDI^+^ cells increased more than 6-fold, compared to uninfected mice (p = 0.04, Fig. 4C).

Further, we asked whether recombinant D4GDI can reduce bacterial burden in *M*. *tb*-infected mice. We infected C57BL/6 wild type mice with *M*. *tb* H37Rv by aerosol. Some infected mice were given recombinant D4GDI (10 ng/ml) through tail vein injection 0, 4, 8, 12, 16, 20, 24 and 28 days after infection. Thirty days later, we measured CFU in the lungs. Recombinant D4GDI reduced CFU by 4 fold (p = 0.0001, Fig. 4D). These findings suggest that D4GDI produced by *M. tb*-expanded CD4^+^CD25^+^Foxp3^+^ T-cells inhibits *M. tb* growth in mice.

### Apoptotic Foxp3^+^ cells are a source of D4GDI

**Figure 5:**
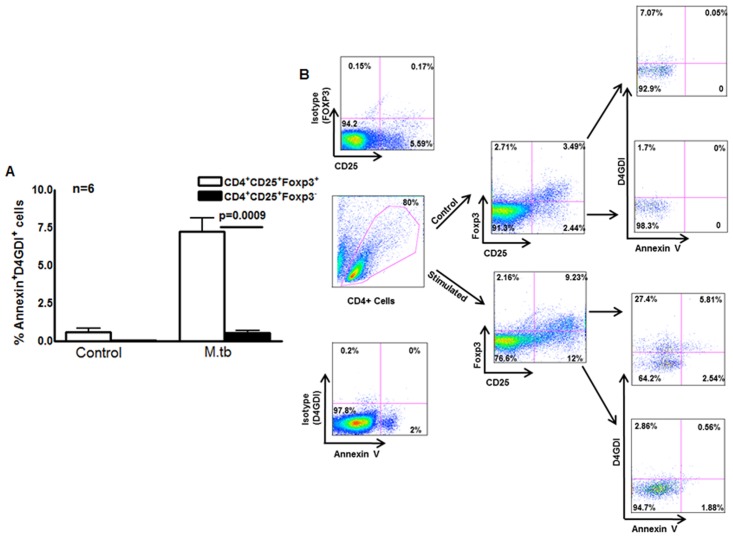
Apoptotic Foxp3^+^ cells are the source for D4GDI. (A) Expression of D4GDI by apoptotic Foxp3^+^ cells. CD4^+^ cells and autologous monocytes from 6 persons with LTBI were cultured with γ-irradiated H37Rv. After 4 days, CD4^+^CD25^+^Foxp3^+^, CD4^+^CD25^+^Foxp3^-^ and CD4^+^CD25^-^Foxp3^-^ cells that expressed annexinV and/or D4GDI were identified by flow cytometry. Mean values, p values and SEs are shown. (B) A representative flow cytometry plot is shown.

Our findings above suggest that T-cell supernatants contain D4GDI, which inhibits growth of *M. tb* in MDMs (Fig. 1 and S1 Fig.). However, in humans, D4GDI is expressed primarily by T and B cells [15] and is not a secreted protein. We asked whether D4GDI is released when terminally differentiated Foxp3^+^ cells undergo apoptosis. CD4^+^ cells and autologous monocytes from 6 persons with LTBI were cultured with γ-irradiated *M*. *tb* H37Rv. After 4 days, annexinV expression by CD4^+^CD25^+^Foxp3^+^, CD4^+^CD25^+^Foxp3^-^ and CD4^+^CD25^-^Foxp3^-^ cells was determined by flow cytometry. Among CD4^+^ cells exposed to γ-irradiated *M. tb*, 7.2 ± 1.9% of CD25^+^FoxP3^+^D4GDI^+^, 4.7 ± 0.3% of CD25^+^FoxP3^+^D4GDI^-^ and 1.5 ± 0.6% of CD4^+^CD25^+^ FoxP3^-^D4GDI^-^ cells are annexinV^+^ (Figs. 5A and C). As an alternative measure of apoptosis, we determined caspase 3 expression in cultured cells. Similar to the above findings, among CD4^+^ cells exposed to γ-irradiated *M. tb*, 15.8 ± 3.3% of CD25^+^Foxp3^+^D4GDI^+^ and 0.82 ± 0.1% of CD25^+^Foxp3^+^D4GDI^-^cells were caspase 3^+^ (S3 Fig.). Among CD4^+^CD25^+^Foxp3^-^ cells 3.5 ± 2.1% of them were caspase 3^+^ (S3 Fig.) and none were D4GDI^+^, suggesting that only a subpopulation of CD4^+^Foxp3^+^D4GDI^+^ cells that undergo apoptosis release D4GDI. To further determine if D4GDI is specifically released by apoptotic CD4^+^Foxp3^+^ cells but not by CD4^+^Foxp3^-^ cells, we cultured the latter cells in the presence of cyclosporin A (10 μg/ml), a known inducer of CD4^+^ T-cell apoptosis. As shown in S4 Fig., apoptotic CD4^+^Foxp3^-^ cells did not express D4GDI despite induction of apoptosis.

### Macrophages internalize D4GDI

We next determined whether MDMs can take up D4GDI. MDMs from 6 healthy donors were infected with GFP- *M*. *tb* H37Rv as described in the methods, and some of the uninfected and infected MDMs were cultured with N-terminal-tagged GST-D4GDI fusion protein. Using confocal microscopy, as described in the methods, we found significant amounts of internalized D4GDI fusion protein in *M*. *tb* H37Rv-infected MDMs (S5 Fig.).

### Interleukin-1 beta (IL-1β), Tumor Necrosis Factor-alpha (TNF-α) and Granulocyte Colony-Stimulating Factor (G-CSF) contribute to D4GDI-dependent inhibition of *M. tb* growth

**Figure 6:**
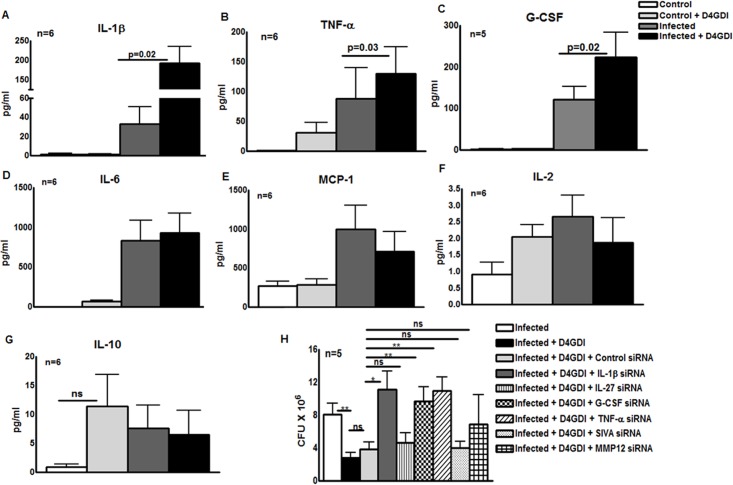
IL-1β and TNF-α contribute to D4GDI-dependent inhibition of *M. tb* growth. MDMs from 5 to 6 healthy donors with LTBI were infected with H37Rv, and control and infected MDMs were cultured with or without recombinant D4GDI. After 6 hr, culture supernatants were collected and levels of 27 different cytokines and chemokines were measured by multiplex ELISA. (A) IL-1β production. (B) TNF-α production. (C) G-CSF production. (D) IL-6 production. (E) MCP-1 production. (F) IL-2production. (G) IL-10 production. (H) Effect of IL-1β, IL-27, G-CSF, TNF-α, SIVA and MMP12 siRNAs on D4GDI-dependent *M. tb* growth inhibition. MDMs from 5 donors were transfected with siRNA for IL-1β, IL-27, G-CSF, TNF-α, SIVA, MMP-12 or scrambled siRNA (control siRNA), and infected with H37Rv at a MOI of 1:2.5 (2.5 *M. tb* to one MDM). Some infected MDMs were cultured with recombinant D4GDI (10 ng/ml). After 7 days, CFU were measured. Mean values, p values and SEs are shown. *p<0.05, **p<0.005.

To identify molecules that control D4GDI-mediated inhibition of *M. tb* growth in MDMs, we cultured *M. tb* H37Rv-infected MDMs from 5 to 6 healthy donors, with or without recombinant D4GDI. After 6 hr, culture supernatants were collected, and levels of 27 different cytokines and chemokines were measured, using a multiplex ELISA system. Recombinant D4GDI increased levels of interleukin-1 beta (IL-1β) from 43 ± 33 pg/ml to 193 ± 104 pg/ml (p = 0.02, Fig. 6A), tumor necrosis factor-alpha (TNF- α) from 88 ± 126 pg/ml to 129 ± 111 pg/ml (p = 0.03, Fig. 6B), and granulocyte colony-stimulating factor (G-CSF) from 122 ± 67 pg/ml to 223 ± 135 pg/ml (p = 0.02, Fig. 6C). Recombinant D4GDI had no effect on cytokine production by uninfected MDMs (Fig. 6). To identify additional molecules that are involved in D4GDI-mediated inhibition of *M*. *tb* growth, we compared gene expression profiles of *M. tb*-infected MDMs, cultured with or without recombinant D4GDI, using microarray analysis for 40,000 genes. MDMs from 4 healthy donors were infected with *M*. *tb* H37Rv and some of the infected MDMs were cultured with recombinant D4GDI. After 6 hr, total RNA was extracted from control, infected and D4GDI-treated infected MDMs. In all 4 donors, mRNA expression was >1.25-fold higher for 42 genes <1.25-fold lower for 4 genes in D4GDI-treated cells (S1 Table). Because the focus of our study is to identify the molecules that are involved in D4GDI-dependent inhibition of *M. tb* H37Rv growth, we selected four upregulated genes, IL-1β, TNF-α, MMP-12 and IL-27, for further study, because these molecules have been shown to play important roles in *M. tb* infection. D4GDI induced G-CSF production (Fig. 6C) but we did not find differences in mRNA expression. We also included G-CSF and a downregulated gene called SIVA (proapoptotic molecule) for further study. Transfection of *M. tb*-infected MDMs with IL-1β and TNF-α siRNAs reduced mycobacterial growth inhibition by D4GDI by 80% (p<0.05, Fig. 6H) and G-CSF siRNA decreased D4GDI-induced growth inhibition by 30% (p<0.05, Fig. 6H). In contrast, MMP-12, SIVA and IL-27 siRNAs had no effect on D4GDI-dependent *M*. *tb* H37Rv growth inhibition (Fig. 6H).

### Reactive oxygen species (ROS) contribute to D4GDI-induced IL-1β, TNF-α and G-CSF dependent growth inhibition

**Figure 7:**
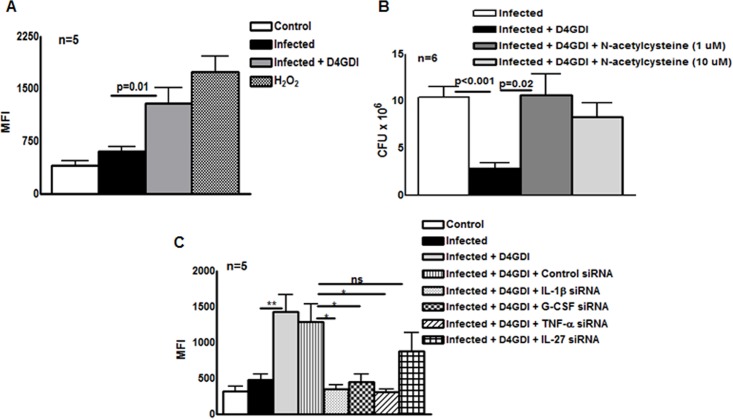
Reactive oxygen species (ROS) contribute to D4GDI-induced IL-1β, TNF-α and G-CSF dependent growth inhibition. MDMs from 5 donors were infected with H37Rv at a MOI of 1:2.5 (2.5 *M. tb* to one MDM). To some wells, recombinant D4GDI (10 ng/ml) or hydrogen peroxide (10 μM) were added. (A) After 24 hr, cells were labeled with 2′,7′-dichlorofluorescein, and ROS expression was measured by flow cytometry. (B) MDMs from 6 donors were cultured. N-acetylacysteine was added to some wells and CFU were measured after 7 days. (C) IL-1β or TNF-α regulate D4GDI-dependent ROS production. MDMs from 5 donors were transfected with siRNA for IL-1β, TNF-α, G-CSF, IL-27or scrambled siRNA (control siRNA), and infected with H37Rv at a MOI of 1:2.5. Some infected MDMs were cultured with recombinant D4GDI (10 ng/ml). After 24 hr, cells were labeled with 2′,7′-dichlorofluorescein, and ROS expression was measured by flow cytometry. Mean values and SEs are shown. *p<0.05, **p<0.005.

Because D4GDI increases production of ROS by mononuclear phagocytes in other experimental systems [15], [16], we wished to determine if D4GDI-induced IL-1β-, TNF-α- and G-CSF-dependent growth inhibition depended on ROS production. ROS production by MDMs was enhanced by infection with *M. tb* H37Rv, and this was further increased by addition of D4GDI (MFI of 1286.8 ± 501.9 vs. 598.4 ± 158.3, p = 0.01, Fig. 7A). ROS levels were similar with addition of D4GDI and hydrogen peroxide (Fig. 7A). To determine if D4GDI-dependent ROS production inhibits growth of *M. tb* H37Rv, we used N-acetylcysteine (NAC), a scavenger of ROS. D4GDI reduced CFU in infected MDMs by 84% and NAC completely abrogated this effect (p = 0.02, Fig. 7B), suggesting that D4GDI inhibits *M. tb* H37Rv growth by inducing *M. tb*-infected MDMs to produce ROS. We also found that transfection of *M. tb*-infected MDMs with IL-1β, TNF-α and G-CSF siRNAs reduced D4GDI-induced ROS production (p<0.05 Fig. 7C).

### D4GDI enhances the apoptosis of *M. tb*-infected macrophages

**Figure 8:**
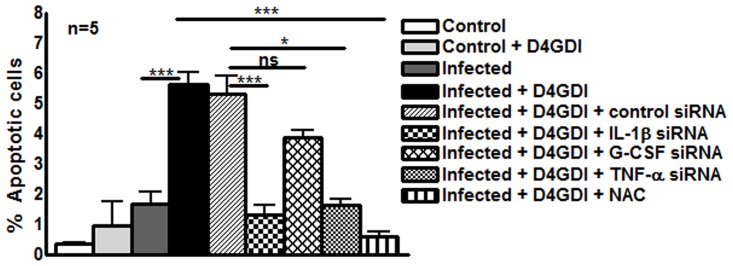
D4GDI enhances apoptosis of *M. tb*-infected MDMs. (A) Apoptosis of control and *M. tb*-infected MDMs in the presence of D4GDI. MDMs from 5 donors were infected with H37Rv at a MOI of 1:2.5 (2.5 *M. tb* to one MDM). To some control and *M. tb*-infected MDM wells, recombinant D4GDI (10 ng/ml) was added. After 3 days, a TUNEL assay was performed to identify apoptotic cells by flow cytometry. MDMs were transfected with siRNA for IL-1β, TNF-α, G-CSF, or scrambled siRNA, and infected with H37Rv at a MOI of 1:2.5 (2.5 *M. tb* to one MDM). Some infected MDMs were cultured with recombinant D4GDI (10 ng/ml). After 3 days, a TUNEL assay was performed to determine the apoptotic cells by flow cytometry. Mean values and SEs are shown. *p<0.05, ***p<0.0005.

D4GDI increased the production of IL-1β, TNF-α, G-CSF and ROS by *M*. *tb* H37Rv-infected MDMs to inhibit *M*. *tb* growth. We next asked whether D4GDI-dependent growth inhibition is due to enhanced ROS-induced apoptosis of infected MDMs. *M*. *tb* H37Rv infection increased the percentage of apoptotic MDMs and this was further increased by addition of D4GDI (5.6 ± 0.9% vs. 1.7 ± 0.9%, Fig. 8). Addition of NAC, a scavenger of ROS, inhibited D4GDI-mediated apoptosis. We also found that D4GDI-induced apoptosis was reduced by transfection of *M. tb*-infected MDMs with siRNAs for IL-1β or TNF-α (p<0.05) but not by G-CSF siRNA (Fig. 8).

### *M. tb*-expanded CD4^+^CD25^+^ (85% Foxp3^+^) cells and D4GDI induce dosR expression by *M. tb*

**Figure 9:**
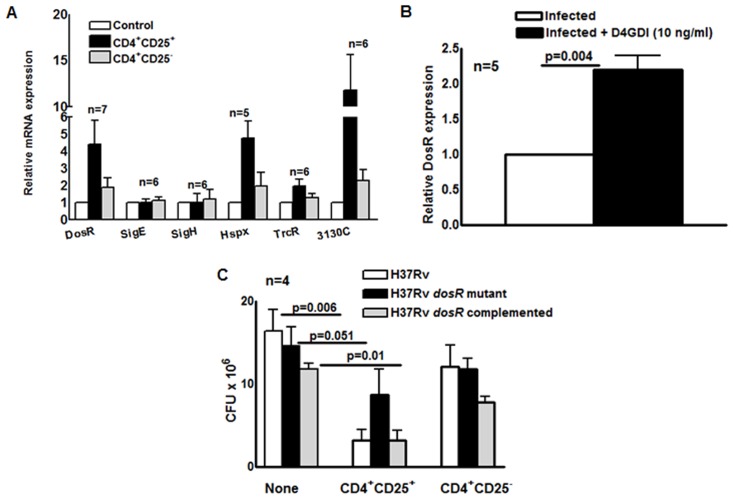
Effect of CD4^+^CD25^+^Foxp3^+^cells and D4GDI on *M. tb* expression of *dosR*. (A) MDMs from 7 donors with LTBI were infected with *M. tb* and cultured with autologous *M. tb*-expanded CD4^+^CD25^+^ (85–90% Foxp3^+^) or CD4^+^CD25^-^ (<5% Foxp3^+^) cells in Transwells. After 7 days, mycobacterial mRNA was quantified by real-time PCR. Mean values and SEs are shown. (B) Effect of D4GDI on *DosR* expression. MDMs from 5 healthy donors were infected with H37Rv, with or without D4GDI (10 ng/ml). After 7 days, *DosR* expression was quantified by real time PCR. ^**^ *P* = 0.004. (C) Effect of CD4^+^CD25^+^FoxP3^+^ cells on the growth of a *M. tb DosR* deletion mutant. MDMs from four donors with LTBI were infected with the H37Rv strains shown, and *M. tb*-expanded CD4^+^CD25^+^ (85–90% Foxp3^+^) cells were added in Transwells, at a T-cell:macrophage ratio of 1:9. After 7 days, CFU were measured. Mean values and SEs are shown. Mean values, p values and SEs are shown.

To characterize the physiologic state of *M. tb* in MDMs during exposure to *M. tb*-expanded CD4^+^CD25^+^Foxp3^+^ T-cells, we used real-time PCR to measure expression of *dosR*, which encodes a transcription factor that is upregulated in response to hypoxia and nitric oxide [17]-[19]. *DosR* activates expression of multiple genes when *M. tb* enters a nonreplicative state, including dormancy induced by anaerobic conditions [20]. MDMs from 7 healthy tuberculin reactors were infected with *M. tb* H37Rv and cultured with autologous *M. tb*-expanded CD4^+^CD25^+^ (85% Foxp3^+^) or CD4^+^CD25^-^ cells in Transwells. After 7 days, *dosR* expression was increased 4-fold in MDMs exposed to CD4^+^CD25^+^ (85% Foxp3^+^) cells (p = 0.04, Fig. 9A), but not in those exposed to CD4^+^CD25^-^Foxp3^-^ cells.

Expression of *Rv3130c* and *hspX*, which are part of the DosR regulon, was also markedly increased in MDMs exposed to CD4^+^CD25^+^ (85% Foxp3^+^) cells (p<0.07, Fig. 9A). Addition of D4GDI to *M. tb*-infected MDMs also induced *dosR* expression two-fold (p = 0.004, Fig. 9B). Because *DosR* can be activated by multiple stresses [21], we evaluated other stress-associated regulators to determine whether D4GDI induced a generalized mycobacterial stress response that was not associated with non-replicating persistence. Expression of these genes (*trcR*, *sigE* and *sigH)* was not increased after exposure to CD4^+^CD25^+^ (85% Foxp3^+^) cells (Fig. 9A).

A *M. tb dosR* deletion mutant showed no growth defect in MDMs, compared to wild type *M*. *tb* H37Rv, but addition of CD4^+^CD25^+^ (85% Foxp3^+^) cells reduced CFU of wild type *M*. *tb* H37Rv by 80%, compared to only 40% for the *dosR* mutant (Fig. 9C). The *dosR* complemented strain behaved similarly to wild type *M*. *tb* H37Rv, confirming that this difference was due to *dosR* and not to effects on adjacent *M. tb* genes. These findings demonstrate that *M. tb*-expanded CD4^+^CD25^+^Foxp3^+^ T-cells and D4GDI inhibit intracellular mycobacterial growth through mechanisms that upregulate expression of *dosR*, and may induce *M. tb* to enter a state of non-replicating persistence.

### D4GDI expression by TB patients

**Figure 10:**
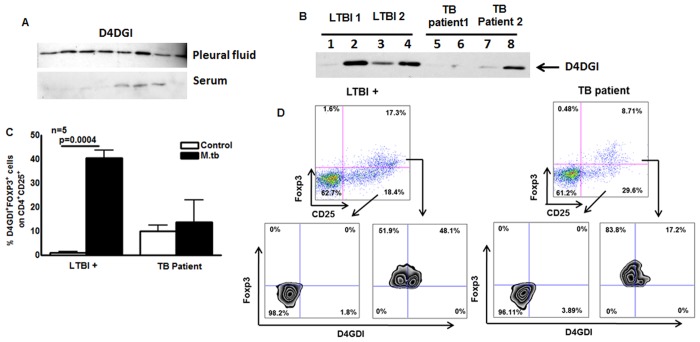
D4GDI expression by TB patients. (A) D4GDI is highly expressed in pleural fluid of TB patients. Pleural fluid and serum from 10 patients were collected, and equal amounts of protein were subjected to Western blotting with a monoclonal antibody to D4GDI. Data from 8 patients are shown. (B) PBMC from TB patients express reduced amounts of D4GDI. PBMC from 5 donors with LTBI and 5 TB patients were cultured with γ-irradiated H37Rv (10 μg/ml). After 5 days, culture supernatants were subjected to Western blotting with monoclonal antibody to D4GDI. Data from 2 patients and 2 individuals with LTBI+ are shown. (C) Intracellular staining for FOXP3^+^D4GDI^+^ cells. CD4^+^ cells from PBMC of 5 donors with LTBI+ and 5TB patients were cultured with autologous monocytes in medium alone, or with γ-irradiated H37Rv (10 μg/ml). After 4 days, immunolabeling and flow cytometry were performed. (D) A representative flow cytometry plot is shown. Mean values, p values and SEs are shown.

To investigate the potential contribution of D4GDI *in vivo* at the site of disease, we studied patients with tuberculous pleuritis, as the pleural immune response usually leads to clearing of local infection [22], [23]. Western blotting showed that D4GDI expression was significantly higher in pleural fluid than in serum of 10 patients with tuberculous pleuritis (Fig. 10A), suggesting that D4GDI is part of an effective local human immune response to *M. tb* infection.

In contrast to tuberculous pleurtis, systemic immunity is reduced in patients with pulmonary TB, whose PBMC show decreased production of IFN-γ in response to mycobacterial antigens, compared to healthy persons with LTBI, who have protective immunity to *M. tb* [8]. We stimulated PBMC from TB patients and donors with LTBI with γ-irradiated *M. tb*, and measured D4GDI levels in culture supernatants by Western blotting. Expression of D4GDI was significantly reduced in TB patients, compared to findings in donors with LTBI+ (Fig. 10B). When CD4^+^ cells from 5 TB patients and 5 donors with LTBI+ were cultured with autologous monocytes and γ-irradiated *M. tb* for 4 days, the percentages of CD4^+^CD25^+^Foxp3^+^D4GDI^+^ cells were higher in persons with LTBI+ (p = 0.0004, Fig. 10C) compared to TB patients, suggesting reduced expansion of D4GDI^+^ cells in TB patients.

## Discussion

In the current study we found that human CD4^+^CD25^+^ (85% Foxp3^+^) cells inhibit growth of *M. tb* in macrophages, providing the first evidence that CD4^+^CD25^+^ (85% Foxp3^+^) cells contribute to effective immunity against an intracellular pathogen. These salutary effects were mediated through production of D4GDI, which enhanced apoptosis of MDMs to inhibit *M. tb* growth through the production of IL-1β, TNF-α and reactive oxygen species. D4GDI was concentrated at the site of disease in tuberculosis patients, D4GDI^+^ cells were expanded in mice infected with *M. tb*, and recombinant D4GDI inhibited *M. tb* growth in mice, suggesting that this molecule contributes to immune defenses *in vivo*. Furthermore, CD4^+^CD25^+^ (85% Foxp3^+^) cells and D4GDI upregulated the mycobacterial gene *DosR* which activates expression of multiple genes when *M. tb* enters a nonreplicative state. The sum of these data suggest a novel role for human CD4^+^CD25^+^Foxp3^+^ cells in protection against infection and identify a new molecule, D4GDI, through which such protection is mediated.

Tregs influence the immune response to parasitic, bacterial, viral and fungal pathogens. The effects may be favorable or harmful to the host, depending on the pathogen and stage of infection. In *Leishmania* infection, Tregs persist at the site of infection, are essential for parasite persistence but favor host immunity to exogenous reinfection [24], [25]. In human *Plasmodium falciparum* infection, the percentage of Tregs correlates directly with parasitic growth [26] and depletion of Tregs enhances survival of mice infected with *P. yoelii* [27], indicating the negative effects of Tregs on the host in malaria. In contrast, Tregs suppress *Helicobacter pylori*-specific T cell responses [28], reducing gastric inflammation and ulceration [29]. Tregs negatively affect host immune responses to viral infections, such as HIV [30], cytomegalovirus [31], Epstein-Barr virus and hepatitis B and C [32]–[35]. We found that a subpopulation of CD4^+^CD25^+^Foxp3^+^ T-cells enhance immunity by producing D4GDI, which inhibits *M. tb* growth in human macrophages. Our studies are further supported by recent findings that in *Candida albicans* infected mice, Tregs enhance Th17 cytokine production to clear the fungal infection [36]. Recent studies have shown that human CD4^+^CD25^+^ cells express Foxp3 transiently and do not have classical regulatory function. Previously we found that *M. tb*-expanded CD4^+^CD25^+^Foxp3^+^ cells produce IL-10 and TGF-β to inhibit IFN-γ production by T-cells [6], [7]. Our findings in Fig. 2D indicate that the subpopulation of CD4^+^CD25^+^Foxp3^+^ cells that express D4GDI do not express IL-10 and TGF-β, suggesting that they are not immunosuppressive. Our current findings suggest that subpopulations of *M. tb*-expanded human Tregs differ in their function.

D4GDI is one of the Rho GDP dissociation inhibitors, small GTP-binding proteins that stabilize the GDP dissociation inhibitor-bound form of Rho GTPases, sequestering Rho GTPases at the plasma membrane [15], [37]. In humans, D4GDI is expressed primarily by T and B cells [15] and is involved in diverse cellular functions, including proliferation, cell signaling, cytoskeletal organization and apoptosis [15], [38], but there is limited information available on the role of D4GDI in microbial infection. Overexpression of D4GDI in T-cells reduces HIV replication through inactivation of RhoA, which reduces viral receptor clustering and lowers the efficiency of virus-cell membrane fusion [39]. However, given the differences in intracellular replication strategies of HIV and *M. tb*, this mechanism would not explain our current results.

We found that silencing of IL-1β and TNF-α gene expression reduces D4GDI-dependent inhibition of *M. tb* growth (Fig. 6). IL-1αβ^-/-^ and IL-1R^-/-^ mice are highly susceptible to *M. tb* infection [40], [41] and IL-1β inhibits *M. tb* growth in macrophages through caspase-3 activation [42], suggesting an important role for IL-1β in innate immune responses against *M. tb* infection. In humans, *M. tb* induces IL-1β production through its interaction with TLR2, TLR6 and NOD2 receptors [43]. In addition, TLR2/1-induced antimicrobial responses against *M. tb* depend on convergence of the IL-1β and vitamin D receptor activation pathways [44], [45]. In mice, caspase-1-independent IL-1β production is critical for host resistance to *M. tb* but does not require TLR signaling [41]. Similarly, TNF-α is essential for control of infection with *M. tb* in animals [46] and inhibits growth of *M. tb* in human monocytes and alveolar macrophages [11], [47], [48]. IL-1β inhibits *M. tb* growth in macrophages by enhancing TNF-α production and through caspase-3 activation [42], suggesting the vital role of these two molecules in *M. tb* infection. In the current study we found that a subpopulation of Tregs produce a novel molecule, D4GDI, which induces IL-1β and TNF-α production by human macrophages to inhibit *M. tb* growth.

We found that IL-1β and TNF-α produced by MDMs in response to D4GDI enhance ROS production, and inhibition of ROS reverses D4GDI-dependent inhibition of *M. tb* growth in macrophages (Fig. 7). ROS are vital in eliminating many pathogens, including *M. tb* [49], [50] and patients with chronic granulomatous disease, whose phagocytic cells fail to generate superoxide, are highly susceptible to infections with fungi and bacteria, including mycobacteria [51], [52]. D4GDI is recruited to the phagosome of RAW macrophages after infection with *Listeria monocytogenes* [53], but its role in ROS production is controversial. Some investigators concluded that D4DGI inhibited ROS production in response to *Listeria* [53], whereas others showed that phagocytic cells from D4GDI-deficient mice produce less superoxide [15], [16]. Our current findings suggest that D4GDI produced by CD4^+^CD25^+^Foxp3^+^ T-cells increases IL-1β and TNF-α production which enhance ROS production by human macrophages in response to *M. tb* infection, reducing bacillary replication. ROS can induce apoptosis, enhance autophagy and elicit production of antimicrobial peptides in *M. tb*-infected macrophages [54], [55], all of which can inhibit intracellular mycobacterial growth [56]–[58].

We found that ROS generated by D4GDI increase *M. tb*-infected macrophage apoptosis and mycobacterial killing (Fig. 8). Apoptosis is a potent mechanism for elimination of mycobacteria. More virulent strains of the *M. tb* complex inhibit apoptosis and grow more rapidly in macrophages, whereas attenuated strains such as H37Ra and BCG elicit greater apoptosis and show reduced intracellular growth [59], [60]. The *M. tb nuoG* gene inhibits macrophage apoptosis and a *nuoG* deletion mutant has reduced virulence in SCID mice [61]. ROS generated during *M. tb* infection induce signaling molecules, such as signal-regulating kinase, which induce apoptosis [62]. We found that D4GDI enhances apoptosis of *M. tb* infected macrophages through the production of IL-1β, TNF-α and ROS to inhibit *M. tb* growth.

Disease manifestations in human TB result not only from uncontrolled bacillary proliferation, but also from tissue inflammation due to a vigorous immune response. In this setting, the role of Tregs in the pathogenesis of TB remains controversial. Elegant experiments in mice suggest that Tregs inhibit immunity to *M. tb* by delaying trafficking of effector T cells to the site of infection [5], [63], but studies in macaques show that the frequency of Tregs is higher in animals that developed LTBI than in those that developed active disease, suggesting that Tregs do not cause progression to active disease [64]. Tregs are well known to downregulate immune responses in many experimental models, and CD4^+^Foxp3^+^ cells expand in response to inflammation during early TB in macaques [64], suggesting that they limit tissue destruction. We found that CD4^+^CD25^+^Foxp3^+^ cells that expand upon exposure to *M. tb* produce D4GDI, which inhibits mycobacterial growth in macrophages. D4GDI was selectively concentrated in pleural fluid of patients with tuberculous pleuritis (Fig. 10A), who have an effective immune response that clears local infection. In addition, PBMC from TB patients with ineffective immunity have reduced production of D4GDI (Fig. 10B). Furthermore, recombinant D4GDI reduced the bacillary burden in *M. tb* infected mice (Fig. 4B). Because CD4^+^Foxp3^+^ cells are enriched in pleural fluid of TB patients [65] and in mycobacterial granulomas [66], we speculate that CD4^+^CD25^+^ cells that express Foxp3 transiently at the site of disease release D4GDI, which inhibits *M. tb* growth. The sum of our studies in humans and murine models of infection suggest that D4GDI produced by CD4^+^CD25^+^Foxp3^+^ T-cells has a previously undescribed positive effect on immunity by enhancing host antimicrobial activity. Our results suggest that D4GDI is part of an effective local human immune response to *M. tb* infection in LTBI individuals. Previous studies have found increased Foxp3^+^cells at the site of TB disease [65], [67]. We found that apoptotic CD4^+^CD25^+^Foxp3^+^ cells express D4GDI and that high D4GDI levels are present at the site of disease, suggesting high turnover of CD4^+^CD25^+^FoxP3^+^ cells at the site of active disease. Further studies to understand the mechanisms that induce D4GDI^+^ cell expansion, production and turnover will provide new insight into the pathogenesis of TB, and identify strategies to enhance immune responses in TB patients.

We found that D4GDI causes intracellular *M. tb* to increase expression of *DosR* and *DosR*-regulated genes (Fig. 9). *DosR* controls expression of a suite of genes required during dormancy induced by anaerobic conditions, when *M. tb* enters a hypometabolic state with minimal replication [20], [68], [69]. *DosR* is activated by two sensor kinases, *DosT* and *DosS*, that fine-tune the response to changes in oxygen, nitrous oxide and carbon monoxide levels, and that appear to modulate the entry of *M. tb* into dormancy in a step-wise fashion [19], [20], [70]. Further work is required to determine the conditions that induce the *DosR* regulon following macrophage exposure to D4DGI. Mycobacterial growth inhibition by CD4^+^CD25^+^Foxp3^+^ cells was reduced in the *M. tb dosR* deletion mutant (Fig. 9B), suggesting that the growth inhibition depends in part on activation of the *DosR* regulon. Our results suggest that macrophage exposure to D4GDI triggers conditions that induce *DosR*, followed by entry of the bacteria into a nonreplicative state. These findings may aid in understanding the factors that induce LTBI.

In summary, our study demonstrates that D4GDI inhibit growth of *M. tb* in macrophages, providing the first evidence that D4GDI contribute to effective immunity against an intracellular pathogen. We are currently determining whether D4GDI can be used as an immunologic marker to detect persons at greatest risk of progression of LTBI to TB, so that this subpopulation can be targeted to receive isoniazid, reduce TB morbidity and improve public health. Additional studies are needed to determine if D4GDI can be used as an immunostimulatory treatment for TB and other infectious diseases.

## Materials and Methods

### Patient population

After obtaining informed consent, blood was obtained from 20 healthy persons with positive QuantiFERON-TB Gold tests, indicative of LTBI, 12 healthy persons with negative QuantiFERON-TB Gold tests and 15 HIV-seronegative patients with culture-proven TB who had received antituberculosis therapy for <4 weeks. All donors were between the ages of 18 and 65. Individuals with LTBI did not have a history of TB or HIV infection, and were not receiving therapy with immunosuppressive drugs.

Pleural fluid was obtained from 10 patients with tuberculous pleuritis who had received antituberculosis therapy for <5 days. All patients had unilateral exudative effusions, and the diagnosis was confirmed by culture of *M. tb* from pleural fluid or tissue, or by histologic evidence of granulomatous pleuritis, combined with a response to antituberculosis therapy.

### Animals

All animal studies were performed on specific-pathogen-free 4–6-week-old female C57BL/6 mice and approved by The Institutional Animal Care and Use Committee of the University of Texas Health Science Center at Tyler.

### Ethics statement

All studies were approved by the Institutional Review Boards of the University of Texas Health Science Center at Tyler (Protocol #640) and the University of Southern California School of Medicine. All the human subjects were in between 18 to 65 years and written informed consent was obtained before enrolling in the study. The Institutional Animal Care and Use Committee of the University of Texas Health Science Center at Tyler approved the animal work (Protocol #488). All animal procedures involving the care and use of mice were in accordance with the guidelines of NIH / OLAW (Office of Laboratory Animal Welfare).

### *M. tb* strains

H37Rv, a virulent laboratory strain of *M. tb*, was used in most experiments. A *dosR* mutant, its complemented strain, and its parental strain of H37Rv, were used in some experiments [68].

### Abs and other reagents

For flow cytometry, we used FITC anti-CD4, allophycocyanin (APC) anti-CD4, APC anti-CD25, PE anti-Foxp3, PE-Cy5 anti-Foxp3 (all from eBioscience), FITC anti-CD14, FITC anti-CD8, PE anti-PD1, FITC anti-D4GDI (Pierce), PE anti-IL-10, PE anti-TGF-β and PE anti-IFN-γ and PE anti-CD127 (e-Bioscience), APO-Direct TUNEL kit (e-Bioscience), Caspase-3 staining kit (BD Pharmingen) and DCFDA—Cellular Reactive Oxygen Species Detection Assay Kit (Abcam). γ-irradiated *M. tb* H37Rv was obtained from BEI Resources. In some experiments recombinant D4GDI (Cell Sciences) and cyclosporin A (Sigma) were used. For neutralization experiments, anti-IFN-γ (BD Pharmingen) and anti IL-22 (e-Biosciences) antibodies were used.

### Isolation of *M*. *tb*-expanded CD4^+^CD25^+^Foxp3^+^ and CD4^+^CD25^-^Foxp3^-^ cells

PBMC were isolated by differential centrifugation over Ficoll-Paque (Amersham Pharmacia Biotech). CD4^+^ and CD14^+^ cells were isolated by positive immunomagnetic selection (MiltenyiBiotec), and were >95% CD4^+^ and CD14^+^, respectively, as measured by flow cytometry. CD4^+^ cells were cultured in 12-well plates at 2 ×10^6^ cells/well in RPMI 1640 containing 10% heat-inactivated human serum, with 2 × 10^5^ autologous CD14^+^ monocytes/well and γ-irradiated *M. tb* (10 µg/ml) at 37°C. After 4 days, CD4^+^CD25^+^ and CD4^+^CD25^-^ cells were isolated, using the Treg isolation kit (MiltenyiBiotec), as described [11]. Eighty five-90% of isolated CD4^+^CD25^+^ cells expressed Foxp3, and <1% of CD4^+^CD25^-^ cells were Foxp3^+^, as determined by intracellular staining.

### Immunolabeling of intracellular Foxp3, IL-10, TGF-β, IFN-γ and D4GDI

For surface staining, 10^6^ cells were resuspended in 100 μl of staining buffer (PBS containing 2% heat-inactivated FBS) and Abs. Cells were then incubated at 4°C for 30 min, washed twice and fixed in 1% paraformaldehyde, before acquisition using a FACS Calibur (BD Biosciences). In some experiments, intracellular staining for Foxp3, IL-10, TGF-β, IFN-γ and D4GDI was performed, according to the manufacturer’s instructions. Controls for each experiment included cells that were unstained, cells to which APC-conjugated goat or rat IgG or PE-conjugated rat IgG had been added and cells that were single stained, either for the surface marker or for intracellular molecules. For Foxp3, IL-10, TGF-β and IFN-γ analysis, we gated on CD4^+^ lymphocytes, and determined the percentages of CD25^+^ and Foxp3^+^ cells. For D4GDI, IL-10, TGF-β and IFN-γ analysis, we gated on CD4^+^Foxp3^+^ cells to identify D4GDI^+^ cells.

### Infection of macrophages with *M*. *tb*, and coculture with CD4^+^CD25^+^Foxp3^+^ T-cells

CD14^+^ monocytes (10^6^/well) were plated in 12-well plates in 1 ml of antibiotic-free RPMI 1640 containing 10% heat-inactivated human serum, and incubated at 37°C in a humidified 5% CO_2_ atmosphere for 4 days to differentiate into macrophages. At the same time, CD4^+^ cells and CD14^+^ monocytes were cultured with γ-irradiated *M. tb* for 4 days, and CD4^+^CD25^+^ and CD4^+^CD25^-^ cells were isolated, as outlined above. In some experiments cells were cultured with Cyclosporin A (20μg/ml). MDMs were infected with *M. tb* H37Rv at a MOI of 1:2.5 (2.5 *M. tb* to one MDM), incubated for 2 hr at 37°C, washed to remove extracellular bacilli, and cultured in RPMI 1640 containing10% heat-inactivated human serum. To some wells, CD4^+^CD25^+^ or CD4^+^CD25^-^ cells were added in Transwells or in the same well, at a ratio of 1 CD4^+^ cell:9 MDMs. To some wells anti-IFN-γ or anti-IL-22 or both, or isotype control antibodies were added on days 0 and 3.

Infected macrophages were cultured for 7 days, at which point macrophage viability was >90%. The supernatant was aspirated, and macrophages were lysed. The supernatant was centrifuged to pellet bacteria, and the pellets were added to the cell lysates. Bacterial suspensions were ultrasonically dispersed, serially diluted, and plated in triplicate on 7H10 agar. The number of colonies was counted after 3 weeks.

### Analysis of proteins in T-cell supernatants

CD4^+^ cells and autologous monocytes were cultured with γ-irradiated H37Rv, as outlined above. After 4 days, CD4^+^ CD25^+^ cells (85–90% Foxp3^+^) and CD4^+^CD25^-^ cells (<5% Foxp3^+^) were isolated by immunomagnetic sorting, and cultured in medium overnight. In some experiments, the supernatants were concentrated and 2D gel electrophoresis was performed, followed by LC-MS/MS analysis of differentially expressed proteins (Kendrick Laboratories, Madison, WI). In other experiments, supernatants were collected and 10μg from each sample was separated by 10% SDS-PAGE, transferred to nitrocellulose and probed with Abs to D4GDI or β-actin (Santa Cruz Biotechnology). After washing, the membranes were incubated with HRP-conjugated secondary Ab (Santa Cruz Biotechnology) and binding was detected by ECL (GE Healthcare).

### MTT assay

The effect of recombinant D4GDI on the viability of MDMs was studied in 96-well plates using the MTT cell proliferation assay kit (ATCC). Briefly, after 7 days of D4GDI treatment, 10 µl of MTT reagent was added in the wells and incubated for 3 hr for the development of purple precipitates. Cells were then lysed in 100 μl of detection reagent and the plate was read at 570 nm after 2 hr of incubation. For blank wells, cells were first lysed and then MTT reagent was added.

### Microarray analysis and Multiplex ELISA

H37Rv-infected MDMs were cultured with or without recombinant D4GDI. After 6 hr, culture supernatants were collected and RNA was isolated from MDMs, using TRIzol reagent (Life Technologies), and pooled RNA was converted to cDNA, followed by microarray analysis (UT, Southwestern, Dallas, TX, USA). In the culture supernatants, the following 27 cytokines and chemokines were measured using multiplex ELISA kit (Bio-Rad). The cytokines and chemokines analyzed were IL-1β, IL-1ra, IL-2, IL-4, IL-5, IL-6, IL-7, IL-8, IL-9, IL-10, IL-12 (p70), IL-13, IL-15, IL-17, basic FGF, Eotaxin, G-CSF, GM-CSF, IFN-γ, IP-10, MCP-1 (MCAF), MIP-1α, MIP-1β, PDGF-BB, RANTES, TNF-α, and VEGF.

### RNA extraction and real-time PCR analysis

MDMs were infected with H37Rv at a MOI of 1:2.5 (2.5 *M. tb* to one MDM), as outlined above, and treated with medium alone or recombinant D4GDI. In some experiments, CD4^+^CD25^+^ or CD4^+^CD25^‑^ cells were added in Transwells. After 3 days, RNA was isolated from 10^6^ MDMs, and reverse transcribed, using the Clone AMV First-Strand cDNA synthesis kit (Life Technologies). For analysis of *M. tb* mRNA, host cells were lysed, bacteria were recovered by centrifugation, and then RNA was extracted by bead-beating in Trizol, followed by further purification.

Mycobacterial primer and probe sets were designed with primer express software (Applied Biosystems); probes were labeled with 5’-fluorescein phosphoramidite and 3’-TAMRA. The primers used to amplify human and *M. tb* cDNA are shown in S2 Table. Real-time PCR was performed using the Quantitect SYBR Green PCR kit (Qiagen) in a sealed 96-well microtiter plate (PE Applied Biosystems) on a spectrofluorometric thermal cycler (7700 PRISM, Applied Biosystems). PCR reactions were performed in triplicate as follows: 95°C for 10 min, and 45 cycles of 95°C for 15 s, 60°C for 30 s, and 72°C for 30 s. Expression of human and *M. tb* genes were normalized to the amount of GAPDH and 16S rRNA transcripts in each sample, respectively.

### siRNA

Freshly isolated CD4^+^ cells were transfected with siRNA for D4GDI or scrambled siRNA. The next day, CD4^+^ cells were washed, and cultured with autologous monocytes and γ-irradiated *M. tb* H37Rv. CD4^+^CD25^+^ and CD4^+^CD25^-^ cells were isolated after 5 days. The efficiency of siRNA knockdown was measured by real-time PCR of D4GDI expression.

MDMs were transfected with siRNA for IL-1β, TNF-α, IL-27, G-CSF, SIVA or MMP12, or control siRNA (all from Santa Cruz Biotechnology). The efficiency of siRNA knockdown was measured by real-time PCR to detect the relevant mRNA. Briefly, 10^6^ MDMs were resuspended in 500 µl of transfection medium, and transfected with siRNA (6 pmoles). After 6 hr, an additional 250 µl of 2X RPMI complete medium was added, and cells were cultured overnight in a 24-well plate. The next day, MDMs were washed and infected with H37Rv, as outlined above, and CFU were measured after 3 days.

### Confocal microscopy to determine internalization of D4GDI

Confocal microscopy was performed to detect intracellular D4GDI, as described previously [71]. MDMs on chamber slides (Lab Tek) were uninfected or infected with GFP expressing *M. tb* H37Rv at a multiplicity of infection of 1:2.5 (2.5 *M. tb* to one MDM) for 2 hr, washed thoroughly, and cultured overnight with GST-D4GDI. After overnight incubation, for intracellular staining, cells were first fixed in 2% paraformaldehyde in PBS (pH 7.2), permeabilized and nonspecific binding was blocked by incubating in blocking buffer. Cells were then incubated overnight with goat polyclonal anti-GST (5 mg/ml) in blocking buffer. As a control, monocytes were incubated with either blocking buffer alone or isotype control antibody (5 mg/ ml). Then, cells were stained with secondary antibody and DAPI (Molecular Probes) to identify the primary signal and nuclei. The cells were washed and mounted with aqueous gel mounting media (Biomedia) containing antifading agent. Confocal images were obtained, using an LSM 510 Meta confocal system (Carl Zeiss) equipped with an inverted microscope (Axio Observer Z1; Carl Zeiss). Immunostained cells were viewed through a Plan-APOCHROMAT 633/1.4 NA oil objective lens, with 2.53 digital magnification, to detect green fluorescence (GFP-expressing *M. tb* H37Rv), blue (DAPI) and red fluorescence (GST-D4GDI). Images were acquired with Zen 2007 software (Carl Zeiss), and scanned images were exported and processed using Adobe Photoshop version 7.0 software (Adobe Systems).

### Measurement of apoptosis of *M*. *tb*-infected macrophages

The effect of recombinant D4GDI on apoptosis of *M. tb*-infected MDMs was measured by using the APO-Direct TUNEL kit. Briefly, MDMs were infected with *M. tb* H37Rv at a MOI of 1:2.5 (2.5 *M. tb* to one MDM). Some MDMs were cultured with D4GDI (10ng/ml). After 72 hr, cells were isolated using trypsin-EDTA and fixed in 1% paraformaldehyde and 70% ethanol in PBS. After overnight incubation at -20°C, cells were washed in washing buffer, resuspended in 50 μl of DNA labeling solution (provided in the kit) and incubated at 37°C. After 60 min, cells were washed twice in rinsing buffer and 500 μl of propidium iodide/RNAse A solution was added and incubated in the dark. After 30 min, cells were analyzed by FACS.

### Infection of mice with *M*. *tb*

Wild type C57Bl/6 mice were infected with H37Rv, using an aerosol exposure chamber, as described previously [72], [73] to deposit ~50–100 bacteria in the lungs. To measure lung CFU, serially diluted lung homogenates were plated on 7H11 agar, supplemented with OADC. CFU were enumerated after 14–22 days incubation at 37°C.

### Statistical analysis

Results are shown as the mean ± SE. Comparisons between groups were performed by a paired or unpaired *t* test, as appropriate.

## Supporting Information

S1 Fig γ-irradiated *M. tb* H37Rv-expanded CD4^+^CD25^+^Foxp3^+^cells produce D4GDI. CD4^+^ cells and autologous monocytes from 6 persons with LTBI were cultured with γ-irradiated *M. tb* H37Rv. After 4 days, CD4^+^CD25^+^ (85–90% Foxp3^+^) and CD4^+^CD25^-^ (<5% Foxp3^+^) cells were isolated by immunomagnetic sorting, and cultured overnight in serum-free medium. The supernatants were pooled and concentrated, and proteins in the supernatants were resolved by 2D gel electrophoresis. Arrows shown are markers. Proteins 1, 2, 3 and 4 in the marked gel of CD4^+^CD25^-^ cells and number 5 in the marked gel of CD4^+^CD25^+^FoxP3^+^ cells were differentially expressed by these cell subpopulations. All 5 bands were cut from the gel and analyzed by LC MS/MS. Proteins 1 through 4 were cytoskeletal proteins and protein 5 was D4GDI.
(TIF)

S2 Fig D4GDI is not toxic to MDMs. 5 × 10^4^ MDMs in 96-well plates were cultured with different concentrations of D4GDI for 7 days and viability was determined by the MTT assay. Mean values and SEs of 3 independent experiments are shown.
(TIF)

S3 Fig Apoptotic CD4^+^Foxp3^+^ cells are the source for D4GDI. (A) Expression of D4GDI by apoptotic Foxp3^+^ cells. CD4^+^ cells and autologous monocytes from 6 persons with LTBI were cultured with γ-irradiated H37Rv. After 4 days, CD4^+^CD25^+^Foxp3^+^, CD4^+^CD25^+^Foxp3^-^ and CD4^+^CD25^-^Foxp3^-^ cells that expressed caspase 3+ and D4GDI were identified by flow cytometry. Mean values, p values and SEs are shown. (B) A representative flow cytometry plot is shown.
(TIF)

S4 Fig Apoptotic CD4^+^Foxp3^-^cells are not the source for D4GDI. (A) Expression of D4GDI by apoptotic CD4^+^cells. CD4^+^ cells and autologous monocytes from 6 persons with LTBI were cultured with Cyclosporine A (20 μg/ml). After 4 days, CD4^+^Foxp3^-^ cells that expressed caspase 3+ and/or D4GDI were identified by flow cytometry. Mean values and SEs are shown. (B) A representative flow cytometry plot is shown.
(TIF)

S5 Fig Macrophages internalize D4GDI. MDM’s from six healthy donors were infected with GFP-H37Rv and some infected MDM’s were cultured with N-terminal-tagged GST-D4GDI fusion protein. After 24 hr, internalization of D4GDI fusion protein was determined by confocal microscopy.
(TIF)

S1 Table The list of up-regulated/down regulated genes in macrophage infected with *M. tb*. Microarray processing and data analysis were performed using Illumina Single Color HumanHT-12_V4. The fold change in expression level of gene was calculated as a normalized mean of infected macrophage+D4GDI/ infected macrophage ratios of the four biological replicates (test sample number) per gene. Genes with >1.25 fold ratios were considered as up-regulated in the test sample.
(DOCX)

S2 Table List of primers used for the study. (DOCX)
