# Supplementary figures and images for: A Rho GDP Dissociation Inhibitor Produced by Apoptotic T-Cells Inhibits Growth of Mycobacterium tuberculosis
Source: PLoS Pathog. 2015 Feb 6;11(2):e1004617. doi: 10.1371/journal.ppat.1004617 (PMC4450061; doi:10.1371/journal.ppat.1004617)

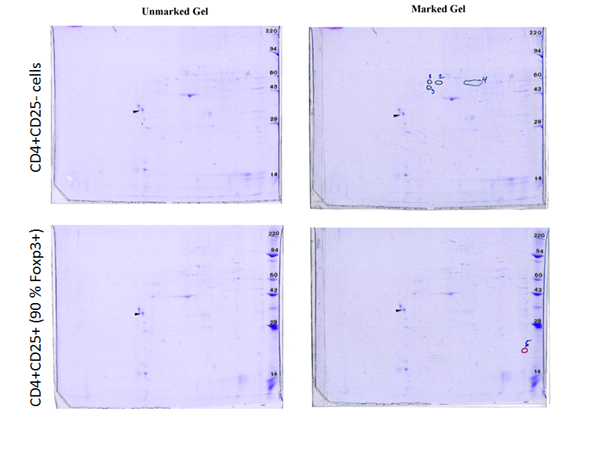

Supplement: S1 Fig — CD4+ cells and autologous monocytes from 6 persons with LTBI were cultured with γ-irradiated M. tb H37Rv. After 4 days, CD4+CD25+ (85–90% Foxp3+) and CD4+CD25- (<5% Foxp3+) cells were isolated by immunomagnetic sorting, and cultured overnight in serum-free medium. The supernatants were pooled and concentrated, and proteins in the supernatants were resolved by 2D gel electrophoresis. Arrows shown are markers. Proteins 1, 2, 3 and 4 in the marked gel of CD4+CD25- cells and number 5 in the marked gel of CD4+CD25+FoxP3+ cells were differentially expressed by these cell subpopulations. All 5 bands were cut from the gel and analyzed by LC MS/MS. Proteins 1 through 4 were cytoskeletal proteins and protein 5 was D4GDI. (TIF) [file ppat.1004617.s001.tif]

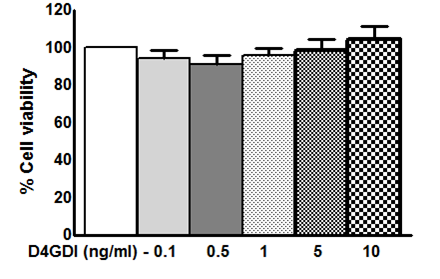

Supplement: S2 Fig — 5 × 10^4^ MDMs in 96-well plates were cultured with different concentrations of D4GDI for 7 days and viability was determined by the MTT assay. Mean values and SEs of 3 independent experiments are shown. (TIF) [file ppat.1004617.s002.tif]

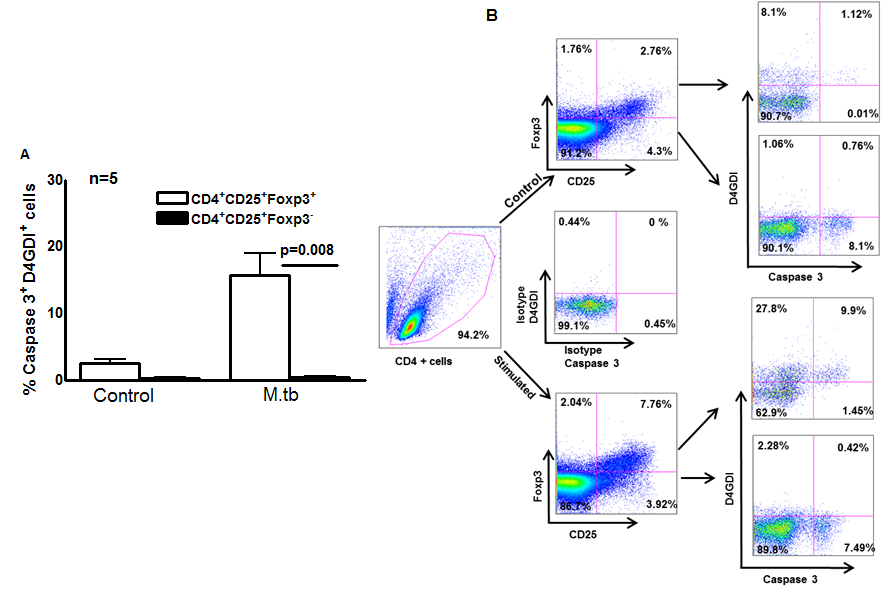

Supplement: S3 Fig — (A) Expression of D4GDI by apoptotic Foxp3+ cells. CD4+ cells and autologous monocytes from 6 persons with LTBI were cultured with γ-irradiated H37Rv. After 4 days, CD4+CD25+Foxp3+, CD4+CD25+Foxp3- and CD4+CD25-Foxp3- cells that expressed caspase 3+ and D4GDI were identified by flow cytometry. Mean values, p values and SEs are shown. (B) A representative flow cytometry plot is shown. (TIF) [file ppat.1004617.s003.tif]

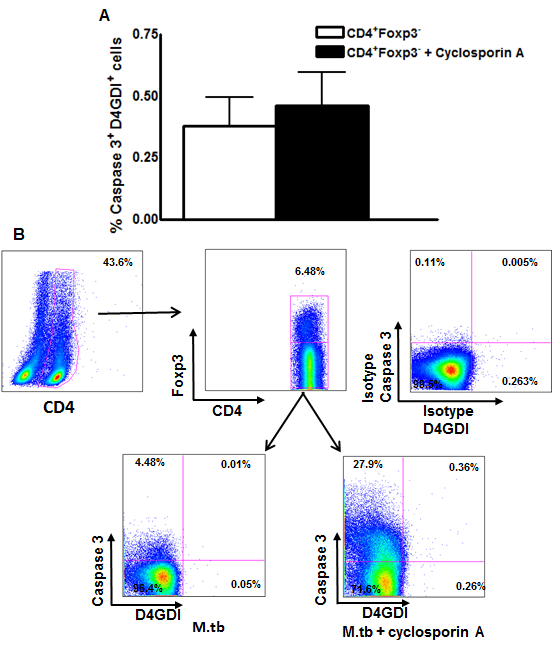

Supplement: S4 Fig — (A) Expression of D4GDI by apoptotic CD4+cells. CD4+ cells and autologous monocytes from 6 persons with LTBI were cultured with Cyclosporine A (20 μg/ml). After 4 days, CD4+Foxp3- cells that expressed caspase 3+ and/or D4GDI were identified by flow cytometry. Mean values and SEs are shown. (B) A representative flow cytometry plot is shown. (TIF) [file ppat.1004617.s004.tif]

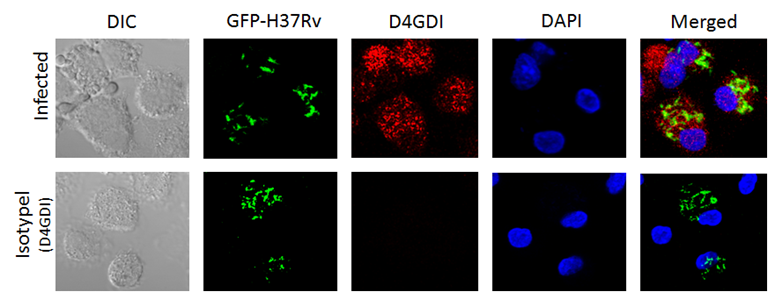

Supplement: S5 Fig — MDM’s from six healthy donors were infected with GFP-H37Rv and some infected MDM’s were cultured with N-terminal-tagged GST-D4GDI fusion protein. After 24 hr, internalization of D4GDI fusion protein was determined by confocal microscopy. (TIF) [file ppat.1004617.s005.tif]
