# Supplementary material for: A Rho GDP Dissociation Inhibitor Produced by Apoptotic T-Cells Inhibits Growth of Mycobacterium tuberculosis
Source: PLoS Pathog. 2015 Feb 6;11(2):e1004617. doi: 10.1371/journal.ppat.1004617 (PMC4450061; doi:10.1371/journal.ppat.1004617)
Supplement: S1 Table — Microarray processing and data analysis were performed using Illumina Single Color HumanHT-12_V4. The fold change in expression level of gene was calculated as a normalized mean of infected macrophage+D4GDI/ infected macrophage ratios of the four biological replicates (test sample number) per gene. Genes with >1.25 fold ratios were considered as up-regulated in the test sample. (DOCX) [file ppat.1004617.s006.docx]

**S1 Table: The list of up-regulated/down regulated genes in macrophage infected with *M. tb***

| GeneBank No. (Reference sequence) | Gene Name | Fold Change | Regulation | Definition |
| --- | --- | --- | --- | --- |
| NM_002426.2 | MMP12 | 2.1526866 | up | Matrix metallopeptidase 12 (macrophage elastase) (MMP12), mRNA. |
| NM_000804.2 | FOLR3 | 1.6951491 | up | Folate receptor 3 (gamma) (FOLR3), mRNA. |
| NM_002981.1 | CCL1 | 1.6614484 | up | Chemokine (C-C motif) ligand 1 (CCL1), mRNA. |
| NM_007115.2 | TNFAIP6 | 1.6313461 | up | Tumor necrosis factor, alpha-induced protein 6 (TNFAIP6), mRNA. |
| NM_000600.1 | IL6 | 1.5830529 | up | Interleukin 6 (interferon, beta 2) (IL6), mRNA. |
| NM_198594.1 | C1QTNF1 | 1.5581161 | up | C1q and tumor necrosis factor related protein 1 (C1QTNF1), mRNA. |
| NM_000575.3 | IL1A | 1.5086963 | up | Interleukin 1, alpha (IL1A), mRNA. |
| NR_001298.1 | HLA-DRB6 | 1.5044574 | up | Major histocompatibility complex, class II, DR beta 6 (pseudogene) (HLA-DRB6), non-coding RNA. |
| NM_002164.3 | INDO | 1.4237986 | up | Indoleamine-pyrrole 2,3 dioxygenase (INDO), mRNA. |
| NM_002164.4 | IDO1 | 1.3912352 | up | Indoleamine 2,3-dioxygenase 1 (IDO1), mRNA. |
| NM_019618.2 | IL1F9 | 1.3864241 | up | Interleukin 1 family, member 9 (IL1F9), mRNA. |
| NM_002438.2 | MRC1 | 1.3598604 | up | Mannose receptor, C type 1 (MRC1), mRNA. |
| NM_022166.3 | XYLT1 | 1.3470085 | up | Xylosyltransferase I (XYLT1), mRNA. |
| NM_002185.2 | IL7R | 1.3295481 | up | Interleukin 7 receptor (IL7R), mRNA. |
| NM_005755.2 | EBI3(IL-27) | 1.3292634 | up | Epstein-Barr virus induced 3 (EBI3), mRNA. |
| NM_001001435.2 | CCL4L1 | 1.3279527 | up | Chemokine (C-C motif) ligand 4-like 1 (CCL4L1), mRNA. |
| NM_002575.1 | SERPINB2 | 1.3112764 | up | Serpin peptidase inhibitor, clade B (ovalbumin), member 2 (SERPINB2), mRNA. |
| NM_003583.3 | DYRK2 | 1.311244 | up | Dual-specificity tyrosine-(Y)-phosphorylation regulated kinase 2 (DYRK2), transcript variant 1, mRNA. |
| NM_175866.2 | UHMK1 | 1.2954242 | up | U2AF homology motif (UHM) kinase 1 (UHMK1), mRNA. |
| NM_052966.2 | FAM129A | 1.2947075 | up | Family with sequence similarity 129, member A (FAM129A), transcript variant 2, mRNA. |
| NM_024430.2 | PSTPIP2 | 1.2937351 | up | Proline-serine-threonine phosphatase interacting protein 2 (PSTPIP2), mRNA. |
| NM_012413.3 | QPCT | 1.2928603 | up | Glutaminyl-peptide cyclotransferase (QPCT), mRNA. |
| NM_021205.4 | RHOU | 1.2909721 | up | Ras homolog gene family, member U (RHOU), mRNA. |
| XM_938742.1 | SGPP2 | 1.2884539 | up | PREDICTED: sphingosine-1-phosphate phosphotase 2 (SGPP2), mRNA. |
| NM_021006.4 | CCL3L1 | 1.2881919 | up | Chemokine (C-C motif) ligand 3-like 1 (CCL3L1), mRNA. |
| NM_031453.2 | FAM107B | 1.2880585 | up | Family with sequence similarity 107, member B (FAM107B), mRNA. |
| NM_005064.3 | CCL23 | 1.2748532 | up | Chemokine (C-C motif) ligand 23 (CCL23), transcript variant CKbeta8-1, mRNA. |
| NM_000675.3 | ADORA2A | 1.2743517 | up | Adenosine A2a receptor (ADORA2A), mRNA. |
| NM_000963.1 | PTGS2 | 1.273359 | up | Prostaglandin-endoperoxide synthase 2 (prostaglandin G/H synthase and cyclooxygenase) (PTGS2), mRNA. |
| NM_015213.2 | DENND5A | 1.2698568 | up | DENN/MADD domain containing 5A (DENND5A), mRNA. |
| XR_018889.1 | LOC650215 | 1.2683591 | up | PREDICTED: similar to Exportin-T (tRNA exportin) (Exportin(tRNA)) (LOC650215), mRNA. |
| NM_001001392.1 | CD44 | 1.2641541 | up | CD44 molecule (Indian blood group) (CD44), transcript variant 5, mRNA. |
| NM_005885.2 | 6-Mar | 1.2639841 | up | Membrane-associated ring finger (C3HC4) 6 (MARCH6), mRNA. |
| NM_000576.2 | IL1B | 1.2631239 | up | Interleukin 1, beta (IL1B), mRNA. |
| NM_002906.3 | RDX | 1.2630762 | up | Radixin (RDX), mRNA. |
| NM_013995.1 | LAMP2 | 1.260982 | up | Lysosomal-associated membrane protein 2 (LAMP2), transcript variant LAMP2B, mRNA. |
| NM_152609.1 | C1ORF71 | 1.2583574 | up | Chromosome 1 open reading frame 71 (C1orf71), mRNA. |
| NM_002994.3 | CXCL5 | 1.2583344 | up | Chemokine (C-X-C motif) ligand 5 (CXCL5), mRNA. |
| NM_000266.1 | NDP | 1.2572687 | up | Norrie disease (pseudoglioma) (NDP), mRNA. |
| NR_003187.1 | NCF1C | 1.2537509 | up | Neutrophil cytosolic factor 1C pseudogene (NCF1C), non-coding RNA. |
| XM_001133190.1 | LOC728835 | 1.2527838 | up | PREDICTED: similar to cytokine, transcript variant 3 (LOC728835), mRNA. |
| NM_005746.2 | NAMPT | 1.2522497 | up | Nicotinamide phosphoribosyltransferase (NAMPT), mRNA. |
| NM_002125.3 | HLA-DRB5 | 7.0471187 | down | Major histocompatibility complex, class II, DR beta 5 (HLA-DRB5), mRNA. |
| NM_006843.2 | SDS | 1.3209146 | down | Serine dehydratase (SDS), mRNA. |
| NM_022827.2 | SPATA20 | 1.2856494 | down | Spermatogenesis associated 20 (SPATA20), mRNA. |
| NM_021709.1 | SIVA | 1.2840258 | down | CD27-binding (Siva) protein (SIVA), transcript variant 2, mRNA |
