# Supplementary material for: A Rho GDP Dissociation Inhibitor Produced by Apoptotic T-Cells Inhibits Growth of Mycobacterium tuberculosis
Source: PLoS Pathog. 2015 Feb 6;11(2):e1004617. doi: 10.1371/journal.ppat.1004617 (PMC4450061; doi:10.1371/journal.ppat.1004617)
Supplement: S2 Table — (DOCX) [file ppat.1004617.s007.docx]

**S2 Table**: List of primers used for the study

| S. No. | Name of the Gene | Primer Sequence |
| --- | --- | --- |
| 1. | *D4GDI* | Forward: AAGTACAAGAAAACGCTGCTGGG  Reverse: TCCAGTAAGGTCCATGGTGATTG |
| 2. | *DosR* | Forward: TTAGCGAGGGCCTGACCA  Reverse: TCACCGTCTTTTCGGCTAGG  Probe: CAAGCAGATCGCCGACCGAATGT |
| 3. | *SigH* | Forward: GGCCTGGCTCTACCGGATA  Reverse: CCGGTTGCCGCTGTTTC  Probe: TGACCAACACCTACATCAACAGCTATCGCA |
| 4. | *SigE* | Forward: TGTTCCGGTCGGTCCAGA  Reverse: GGTGATGCGGTGTAGCCAG  Probe: TTACCAGCCGGGCACCTTCGAA |
| 5. | *HspX* | Forward: ACATTATGGTCCGCGATGGT  Reverse: CGACCGTCGAAGTCCTTCTG  Probe: TGACCATCAAGGCCGAGCGCA |
| 6. | *TrcR* | Forward: CGACATCATGCTTCCCGAT  Reverse: CGTCGGATTCTCGGACCC  Probe: TGGACGGGTTGGAAATCCTGCG |
| 7. | *Rv3130c* | Forward: TGAGCAAGACCGATAACCGTG  Reverse: CAGCGGGTTCTCTTGATCCA  Probe: TTCGTTAATGCTGCCCAACCTGCC |
| 8. | *16S rRNA* | Forward: TCCCGGGCCTTGTACACA  Reverse: CCACTGGCTTCGGGTGTTA  Probe: CGCCCGTCACGTCATGAAAGTCG |
| 9. | *GAPDH* | Forward: GCCATCAATGACCCCTTCATT  Reverse: TTGACGGTGCCATGGAATTT |
| 10 | *IL-1β* | Forward: CACGATGCACCTGTAACGATCA  Reverse: GTTGCTCCATATCCATTTCCCT |
| 11. | *MMP 12* | Forward: ACACCTGACATGAACCGTGA  Reverse: CAATGCCAGATCCAGGTCC |
| 12. | *TNF-α* | Forward: GGAGAAGGGTGACCGACTCA  Reverse: CTGCCCAGACTCGGCAA |
| 13. | *G-CSF* | Forward: GGAGACCTCGTGCCAAATTA  Reverse: TATCTCTGAAGCGCATGGTG |
| 14. | *IL-27* | Forward: CGCTTTGCGGAATCTCAC  Reverse: GGGCATGGAAGGGCTGAA |
| 15. | *SIVA* | Forward: TACAGCTCAAGGTCCGCGTGAGC  Reverse: TCACTGCAGTCCACGAGGCCACA |
